# *Cinchona officinalis* Phytochemicals-Loaded Iron Oxide Nanoparticles Induce Cytotoxicity and Stimulate Apoptosis in MCF-7 Human Breast Cancer Cells

**DOI:** 10.3390/nano12193393

**Published:** 2022-09-28

**Authors:** Laila Naif Al-Harbi, Ghedier M. Al-Shammari, Pandurangan Subash-Babu, Mohammed A. Mohammed, Roaa Ahmed Alkreadees, Abu ElGasim Ahmed Yagoub

**Affiliations:** Department of Food Science and Nutrition, College of Food Science and Agriculture, King Saud University, Riyadh 11451, Saudi Arabia

**Keywords:** *Cinchona officianlis*, bioactive compounds, iron oxide, bioavailability, apoptosis

## Abstract

The present study aimed to synthesize iron oxide nanoparticles loaded with quinine and alkaloids-rich *Cinchona officinalis* (Peruvian bark) stem bark extract, and further evaluate their cytotoxic effect and apoptosis mechanisms in MCF-7 breast cancer cells. Nanoparticles were prepared by biological reduction of iron oxide with *Cinchona officinalis* extract, using the green synthesis method. The nanoparticles were characterized by XRD, FT-IR, and UV-vis spectroscopy and transmission electron microscopy (TEM). In vitro cytotoxicity analyses of *Cinchona officinalis* extract, ferrous oxide, and *Cinchona officinalis* extract-loaded iron oxide nanoparticles (CO-NPs) were carried out using the MTT test for 24 h and 48 h. We found that CO-NPs reduced the MCF-7 cell viability with IC_50_ values of 16.2 and 9 µg/mL in 24 h and 48 h, respectively. In addition, CO-NPs were tested with normal hMSCs to determine their toxicity, and we did not find noticeable cytotoxicity. Confocal fluorescent microscopy revealed that CO-NPs efficiently increased the nuclear condensation and chromatin damage in propidium iodide staining; meanwhile, there was decreased mitochondrial membrane potential in CO-NPs-treated MCF-7 cells. In addition, AO-EB staining confirmed the late apoptotic and apoptotic morphology of cancer cells. Further gene expression analysis confirmed that the upregulation of tumor suppressors, Cdkn1A, Prb, and p53 was significantly increased, and inflammatory traits such as TNF-α and Nf-κb were increased in cancer cells treated with CO-NPs. Apoptotic stimulators such as Bax and caspase-3 expression were highly significantly increased, while mdm-2 and Bcl-2 were significantly decreased. Overall, the enhanced cytotoxic potential of the *Cinchona officianlis* stem bark extract loaded CO-NPs versus free *Cinchona officianlis* extract might be due to the functional stabilization of bioactive compounds, such as alkaloids, quinine, flavonoids, phenolics, etc., into the iron oxide, providing bioavailability and internalization of cinchona metabolites intracellularly.

## 1. Introduction

In 2021, about 2.3 million women had breast cancer [1]. So, it is the most frequent type of cancer worldwide, accounting for 53% of cases from 2010 to 2019 in Saudi Arabia alone [2]. Its most common symptom is feeling an unpainful lump or a tumor in the breast [1]. It extends to other organs via metastasis, spreads quickly, and leads to death. Treatment options for BC include breast removal surgery, radiation therapy, and chemotherapeutic agents [1,3]. However, although these treatments provide temporary relief from pain and extend the length of life, they rarely cure cancer. Also, the effective doses of some of these chemically synthesized drugs were close to toxic levels due to nonspecific effects [3]. Thus, more research should be conducted utilizing in vitro cell models that are specialized for cancer research to identify the cytotoxic mechanisms and the effectiveness of the treatment [4].

Nanotechnology has become popular as a merging point between science and technology. Nanoparticles have special chemical and physical characteristics that allow them to be implemented in different fields, including agricultural, medical, electronic, chemical, and pharmaceutical fields [5]. Metallic nanoparticles, on the other hand, have demonstrated their ability to be used in the medical field by treating various types of cancer. Thus, the biosynthesis of plant-based metallic nanoparticles is considered an advanced technology for curing cancer without affecting healthy cells [5]. The nanosized particles have been proven to facilitate and improve the delivery of drugs into the cells [6]. Nanoparticles can be biosynthesized by various approaches, such as metal and metal oxide, to form functionalized nanoparticles containing many biological structures, such as plant metabolites and other microorganisms, which are less expensive and eco-friendly. Silver nanoparticles, for example, have been shown to initiate the cell cycle, while gold nanoparticles cause apoptosis in cancer cells. In addition, cobalt, nickel, iron, and metal oxide nanoparticles have a superparamagnetic characteristic of the magnetic nano-catalyst which allows them to be used in the biomedical field [5]. In particular, iron oxide nanoparticles are effective at killing cancer cells, with only minor cell death observed in normal human fibroblasts. These findings indicate that iron oxide nanoparticles are safe for treating cancer cells while causing no harm to healthy cells [7].

Plant-based nanoparticles have the potential to cure acute diseases like malaria, HIV, and cancer [8]. *Cinchona officinalis*, Peruvian bark, is a family member of Rubiaceae, originally from South America. The bark is mainly used for medicinal purposes and possesses a strong antimalarial effect. It consists of 16% quinine, 15% alkaloids, 0.25% to 3.0% cinchonidine quinidine, cinchonine, and cinchonine in combination with other vigorous compounds like tannin. In addition, minerals, essential oils, acids, flavonoids, and phytosterols have also been identified. Its medicinal effects include antimicrobial factors, antiarrhythmic, anti-obesity, antioxidant, anti-inflammation, and anticancer properties. Quinine works on cancer cells by inducing apoptosis and preventing cell proliferation depending on dose and duration [9].

In the present study, we synthesized *Cinchona officinalis* extract-loaded iron oxide nanoparticles and examined their effects on cytotoxicity, mitochondrial membrane potential, and gene expression associated with apoptosis mechanisms in MCF-7 breast cancer cells. In addition, the *Cinchona officinalis* iron oxide nanoparticles-induced cell death mechanism has been explored via mitochondrial-dependent and independent mechanistic factor analysis.

## 2. Materials and Methods

### 2.1. Preparation of Cinchona officinalis Bark Methanol Extract

*Cinchona officinalis* stem barks were gathered in Sudan’s Darfur region. The plant was identified by a botanical specialist (King Saud University in Riyadh). After drying, stem barks were crushed using a mortar and pestle and then added to a conical flask containing a 95% methanol solution (solid:solvent ratio of 1:10). After being covered in aluminum foil, the flask was shaken for 6 h (Wrist Action shaker, Burrell Scientific, Pittsburgh, PA, USA). The methanol (Sigma-Aldrich, Inc., St. Louis, MO, USA) was removed using a rotary vacuum evaporator (HAHNVAPOR, HS-2005, Hahn Shin Scientific, Gimpo-si, Korea). The *C. officinalis* stem bark extractive (98.4 mg/mL) was kept for future use.

### 2.2. GC–MS Analysis of the Stem Bark Methanol Extract

A gas chromatography (GC, 7890A) connected to a mass spectrometer (MS, 5975C) was used to determine the bioactive substances in *Cinchona officinalis* stem bark methanol extract (Agilent Technologies, Santa Clara, CA, USA). The column (DB-5MS) had dimensions of 30 m length × 0.25 mm inner diameter × 0.25 μm film thickness. A Triple-Axis detector (MSD), and a 7693 automated liquid sampler were also parts of the analysis system. The system’s electron ionization energy was 70 eV. A 2 μm membrane filter (MF Millipore, Sigma-Aldrich, Inc., St. Louis, MI, USA) was used to filter the extract before injection. For the analysis, 1 μL was injected at 280 °C into the column with a temperature of 300 °C. The mobile phase was helium (1 mL/min).

### 2.3. Biosynthesis of Iron Oxide Nanoparticles

To prepare iron oxide nanoparticles, 0.61 g and 1.22 g of ferric chloride (Sigma-Aldrich, Inc., St. Louis, MI, USA) were separately dissolved in distilled water (75 mL) with constant stirring (200 rpm), resulting in 0.05 and 0.1 M ferric chloride solutions. Then, 0.51 and 1.02 mL, containing 50 and 100 mg, respectively, of the concentrated stem bark methanol extract, were separately diluted in 25 mL of distilled water. At room temperature, an aliquot of 50 mg and 100 mg extract solutions (25 mL) was added dropwise to the iron chloride solution (0.05 and 0.1 M, 25 mL) aided by continuous stirring (200 rpm) for 30 min. The production of iron oxide nanoparticles was revealed by a brownish-black colloidal solution (Figure S1). The synthesized nanoparticles were centrifuged (12,000 rpm, 10 min). The residue was dried at 50 °C and kept at 4 °C until use. Accordingly, 50 mg and 100 mg of the extract each were separately reacted with 0.05 M and 0.1 M of the ferric chloride solution, synthesizing four different iron oxide nanoparticles.

### 2.4. Characterization of Cinchona Officinalis Extract-Loaded Iron Oxide Nanoparticles (CO-NPs)

The formation of iron nanoparticles loaded with *Cinchona officinalis* stem bark methanol extract was monitored by measuring the UV-vis spectra at a wavelength ranging between 200 and 800 nm; a UV-visible spectrophotometer (UV-2450 double-beam, Shimadzu, Tokyo, Japan) was employed. The crystalline phase of the fabricated nanoparticles was examined using an X-ray powder diffractometer (Bruker D8 Advance, Burker UK limited, Coventry, UK). The X-ray powder diffractometer was equipped with a diffracted beam monochromator and a Cu-K radiation source (=1.54 nm; 40 kV; 40 mA). With a scan rate of 0.02°, the scanning of scattered radiation was conducted in the range of 10–90° (2θ). The JCPDS card database was then used to compare the diffraction patterns of the examined nanoparticle samples. An FT-IR spectrometer (Nicolet 6700, Waltham, MA, USA) was used to measure the stem bark’s functional groups from 400 to 4000 cm1 cm^−1^. A transmission electron microscope (TEM) (JEM-1011, JEOL Ltd., Tokyo, Japan) was used to capture morphological images of samples at an acceleration voltage of 160 kV. Before starting the biological experiments, UV radiation was used to sterilize the nanoparticle powder for 10 min to get rid of any potential microbial contaminations during storage. Also, nanoparticles with the predetermined concentration were prepared fresh for experiments.

### 2.5. Cell Lines and Cell Culture Materials

Human breast carcinoma (MCF-7, MDA-MB-231) cells and human mesenchymal stem cells (hMSCs) were obtained from American-type culture collections, (ATCC, Manassas, VA, USA). Fetal bovine serum (FBS), Dulbecco’s modified Eagle medium (DMEM), EDTA, and trypsin were purchased from Gibco, Paisley, UK. The antibiotics, penicillin, and streptomycin were purchased from Hyclone Laboratories, Logan, UT, USA.

#### Cell and Nuclear Staining Agent, cDNA Synthesis Kit and Chemicals

Cell and nuclear staining fluorescent intercalating agents, such as propidium iodide, ethidium bromide, acridine orange, and mitochondrial JC-1 stain were obtained from Sigma (St. Louis, MO, USA). The cell to cDNA synthesis kit and qPCR master mix (SYBR Green/ROX based) were purchased from Qiagen (Hilden, Germany) company. All the fine chemicals for the molecular biology study were purchased from Sigma-Aldrich (St. Louis, MO, USA).

### 2.6. In-Vitro Cytotoxicity Analysis

hMSCs, MDA-MB-231, and MCF-7 cells were plated in cell titer 96-well plates with 1 × 10^4^ cells/mL concentration and allowed to grow for 24 h before treatment with nanoparticles. After 24 h, increasing concentrations of *Cinchona officinalis* extract (COE) and *Cinchona officinalis* extract-loaded iron oxide nanoparticles (CO-NPs) (0, 1.25, 2.5, 5, 10, 20, 40, 80, and 160 µg/mL) were treated then incubated for 24 h and 48 h, respectively. After incubation time, 20 μL of MTT (5 mg concentration of MTT dissolved in 1 mL of phosphate-buffered saline (PBS)) was added to each well in the dark and incubated for 4 h at 37 °C [10]. Then, the developed purple formazan product was centrifuged to sediment. The sediment was dissolved by the addition of 100 μL of DMSO (after removing the supernatant) and absorbance was measured at 570 nm with a 630 nm reference range using a multiwell plate reader (Bio-Rad, Hercules, CA, USA). Quadruplicate assays were carried out for each concentration of COE and CO-NPs with three independent experiments. The percentage of toxicity was calculated using the formula: ((Mean OD of untreated cells − Mean OD of treated cells)/(Mean OD of untreated cells)) × 100. The viability of the cells was expressed as the percent viability of treated cells vs. untreated cells. Tamoxifen and doxorubicin were used to identify the comparative cell growth inhibition with the same IC_50_ concentration, and used as the reference.

### 2.7. Microscopic Studies

#### 2.7.1. Determination of Apoptotic Morphology Using Propidium Iodide Staining

To discriminate apoptotic cells and necrotic cells, MCF-7 cells were grown in 24-well plates, and treated with IC_50_ doses of COE and CO-NPs, such as 24 µg/mL, 8 µg/mL, and untreated MCF-7 cells, and allowed for 48-h incubation. Then, the cells were washed with PBS after removing the media. To stain the treated cell nuclei, 1 mg/mL of propidium iodide (St. Louis, MO, USA) was added and kept in the dark for 20 min at 37 °C. Randomly, 300 stained cells were characterized under an inverted fluorescence microscope at 200× magnification, and the percentage of apoptotic and necrotic morphology was counted manually.

#### 2.7.2. Early and Late Apoptosis Determination Using AO/EtBr Staining

Acridine Orange/Ethidium Bromide (AO/EtBr) dual staining was used to discriminate the hallmark of pre, pro, and late apoptotic morphological alterations as described by [11]. Briefly, human MCF-7 cells were treated with COE and CO-NPs at 24 µg/mL and 8 µg/mL, and untreated control for 48 h in 24-well plates. After 48 h, treated cells were gently rinsed with PBS, then 500 µL of AO/EtBr (1:1, 4 mg/mL) solution was overlayed until the cells became immersed. After 10-min incubation, the dye was gently washed out using PBS rinsing, and the stained cells were immediately observed under an inverted fluorescent microscope (Carl Zeiss, Jena, Germany) fitted with a green filter, and pictures were taken at 200× magnification.

#### 2.7.3. Determination of Mitochondrial Membrane Potential

Mitochondrial biogenesis and functioning are directly associated with the mitochondrial polarization state. The JC-1 staining probe, which normally exists in monomeric form, was primarily applied to determine the mitochondrial membrane potential (Δψm). Cells with polarized or intact mitochondria contain high negative charges, which predominantly convert the positively charged JC-1 monomer into aggregates and emit reddish-orange fluorescence. The cells containing damaged or depolarized mitochondria uptake the JC-1 dye and fluorescence green. MCF-7 breast cancer cells were treated with 24 µg/mL of COE, 8 µg/mL of CO-NPs, and untreated control for 48 h in 24-well plates. After incubation, the experimental cells were gently rinsed with PBS and stained with 5 mM of JC-1 for 10 min at 37 °C. Immediately, the cells containing unbound JC-1 dye were washed with JC-1 washing solution; further, the monomeric green and aggregated red signals were captured using a fluorescent microscope.

### 2.8. Quantitative-Real Time PCR Analysis

Fastlane^®^ Cell cDNA kit (QIAGEN, Hilden, Germany) was used to synthesize directly the cDNA from the treated cells, followed by mRNA expression levels of oxido-redox associated genes (CYP1A, GPx, GSK-3β, TNF-α, and Nf-κb), tumor suppressor genes (Cdkn2A, Rb1, p53, and mdm2), apoptosis-associated genes (Bax, Bcl-2, p21(Cdkn1A), Caspase 3 and Caspase 9, as well as the reference gene GAPDH were quantified using SYBR Green-master mix (QIAGEN, Hilden, Germany) according to the kit instructions. The qPCR was performed using 25 μL reaction volume and 40 cycles were allowed for amplification. The obtained fluorescence from Ct cycles was used to calculate the fold expression levels of targeted genes, and this was compared between untreated control MCF-7 cells, MCF-7 cells treated with COE, and CO-NPs at 24 and 8 µg/mL for 48 h. The relative expression levels were determined by the following formula: ΔΔCt (comparative threshold) = ΔCt (Treated) − ΔCt (Control) [12]. The specific gene expression value was used to plot the gene expressions by calculating 2^−ΔΔCt^. Thus, the gene expression levels were presented as n-fold differences compared to the reference gene.

### 2.9. Statistical Analysis

Statistical analysis of the present experimental data was performed using SPSS software package/version 28.5. The one-way analysis of variance (ANOVA) was used to determine significant variations between the tested groups. Tukey’s test was used to detect significant variations among means at significance levels of *p* ≤ 0.01 and *p* ≤ 0.05. The results were expressed as mean ± SD for six replications in each group.

## 3. Results

### 3.1. Characterization of Cinchona officinalis Stem Bark Extract-Loaded Iron Oxide Nanoparticles (CO-NPs)

CO-NPs were synthesized using 0.05 and 0.1 M concentrations of FeCl_3_ solution and the methanol extract from *Cinchona officinalis* stem barks (50 and 100 mg concentrations were used). Phytochemical compounds, such as polyphenols, flavonoids, glycosides, tannins, etc., all serve as reducing and stabilizing factors for the synthesis of nanoparticles. The interaction of phytochemicals in *Cinchona officinalis* stem bark methanol extract with Fe^3+^ ions resulted in a brownish-black color precipitate (Figure S1), indicating the synthesis of iron oxide nanoparticles [13]. Figure S2A,B shows the UV-vis spectra of the CO methanol extract, iron oxide, and 0.05 M and 0.1 M CO-NPs (50 and 100 mg). The methanol extract and iron oxide each had an absorption peak located at 290 nm, which was lost in CO iron oxide nanoparticles.

**Figure 1:**
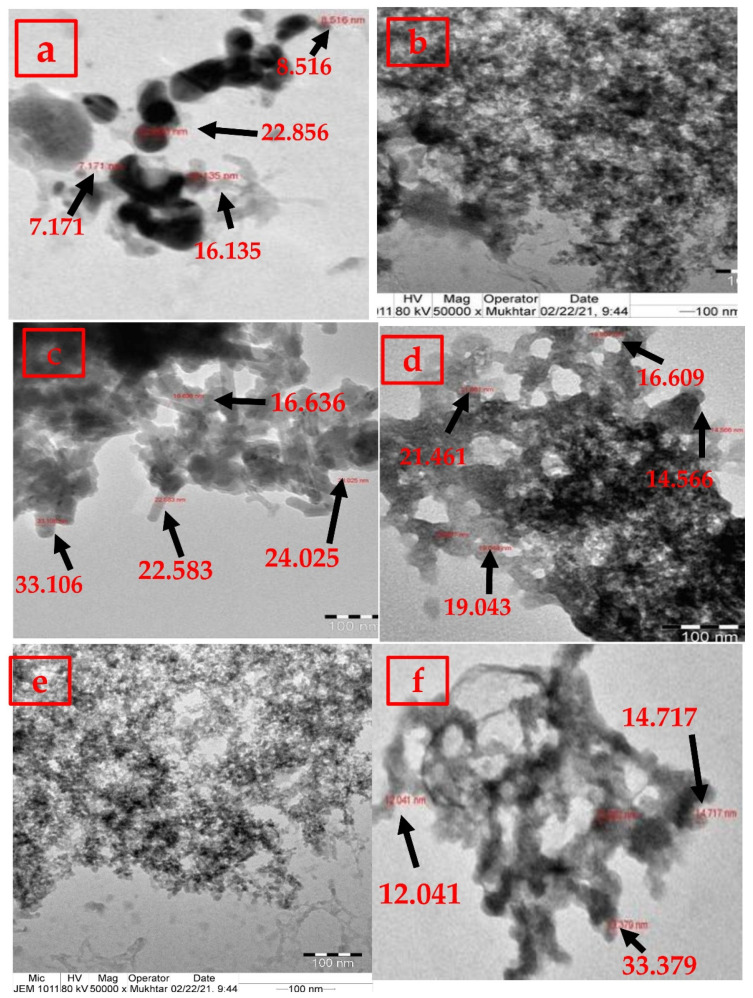
TEM images of 0.05 M and 0.1 M iron oxide (**a**,**b**) and 0.05 and 0.1 M iron oxide nanoparticles prepared using 50 mg (**c**,**d**) and 100 mg (**e**,**f**) of *C. officinalis* stem bark extract.

XRD diffraction peaks of α-Fe-OOH and 50 and 100 mg CO-NPs (0.05 and 0.1 M) are shown in Figure S3a–e. Iron oxide had characteristic diffraction peaks at 2θ = 15.07 (110), 25.41° (120), 33.09° (130, major peak), 35.00° (021), 36.82° (111), 52.07° (211), and 54.56° (221) (JCPDF card no. 29-713). The CO-NPs prepared from the reaction of 50 mg plant extract with 0.05 M and 0.1 M FeCl_3_ had similar diffraction peaks, having characteristic peaks at 2θ values of 37.74° (311), 43.98/44.02° (400), 56.60/56.54° (511), 64.37/64.43° (440), and 75.32/75.56° (533), respectively. These orientation planes were consistent with standard data of 𝛾-Fe_2_O_3_/Fe_3_O_4_ (maghemite/magnetite) diffraction peak PDF card (JCPDS, PDF, File No. 01-071-6336 and 00-039-1346, respectively), with face-centered cubic spinel-like structures [14,15]. Similar results were reported earlier in studying the biosynthesis of iron oxide nanoparticles [16]. On the other hand, 100 mg CO-NPs (0.05 and 0.1 M) displayed diffraction peaks at 2θ values of 31.48/31.7° (104), 35.64/36.23° (110), 41.00/40.29° (113), 48.65/48.98° (024), 54.34/53.71° (116), 62.40/62.45° (214), 63.53/63.49° (300), 75.10/75.32° (220), and 89.47/89.28° (226), respectively. The orientation planes matched with ICDD data file 84-0311, confirming the synthesis of crystalline α-Fe_2_O_3_ (hematite) with a rhombohedral-centered hexagonal structure [13,17]. The 𝛾-Fe_2_O_3_/Fe_3_O_4_ peaks were more intense and narrower in comparison with α-Fe_2_O_3_ peaks, indicating higher crystallinity as a result of the nanoparticles’ agglomeration [18]. The morphology of iron oxide samples is examined by TEM analysis (Figure 1). Both 50 and 100 mg CO-NPs (0.05 and 0.1 M) had needle-like morphologies, with diameters ranging from 16.64 to 33.11 nm and 12.041 to 33.38 nm, respectively. Due to the smaller size range, 100 mg CO-NPs were used in the subsequent cytotoxicity tests.

The FT-IR spectra analysis determines the functional groups loaded into iron nanoparticles. The IR spectra confirmed the presence of several reducing agent functional groups in the methanol extract and ferric oxide nanoparticles (Figure S4a,b). The peak at 3400 cm^−1^ in the CO extract was a result of the O–H bond stretching vibrations of water, phenolic compounds, or carboxylic acids [19]. This peak was seen at 3411 and 3400 cm^−1^, with decreased intensity, in 0.05 M and 0.1 M CO-NPs (50 and 100 mg). This shift in the peak was probably an outcome of the interaction of O–H groups with iron, leading to the generation of iron oxide nanoparticles [20]. The stretching vibrations of C–H in −CH_2_ groups and the asymmetrical stretching vibrations of -C≡C- bonds were responsible for the appearance of the peak at 2927 cm^−1^ [21,22]. In the plant extract, the peak at 1600 cm^−1^, due to stretching of the carboxylic group (COO^−^), was red-shifted to 1615–1621 cm^−1^ in CO-NPs’ spectra [23]. The band at 1444–1517 cm^−1^ in the CO methanol extract was due to the occurrence of amide II groups [24]. Peaks at 1068–1055 cm^−1^ in the CO methanol extract and CO-NPs’ spectra were assigned to C–N stretching vibrations of aromatic and aliphatic amines. In the CO methanol extract, the peaks detected at 766 cm^−1^ (minor) and 611 cm^−1^ (major) were a result of the aromatic C–H bending vibrations of phenolic compounds and aromatic nitriles, respectively. Additionally, the iron oxide’s peaks at 1612 and 3370 cm^−1^ were associated with the surface hydroxides’ O–H bending vibrations, as well as hydrogen bonding and O–H stretching from the water that was adsorbed [19]. Aromatic compounds were present in the methanol extract and confirmed by C=C stretching of aromatic amine at 1377 cm^−1^ that blue-shifted in the nanoparticles [25]. The presence of Fe–O bonds in these samples was assured by peaks at 464–475 cm^−1^ and 611–619 cm^−1^ in Fe-OOH and 50 and 100 mg CO-NPs’ (0.05 and 0.10 M) spectra [13,26]. As can be seen, the IR spectra of the 100 mg CO-NPs exhibited stronger peaks than the corresponding peaks in the 50 mg CO-NPs. The assumption that the functional groups of such bioactive compounds are bonded to the iron oxide surface was corroborated by a change in the spectra shape, a shift in peak, or a change in intensity in the 400–4000 cm^−1^ range.

### 3.2. GC–MS Profile of the Stem Bark Methanol Extract of Cinchona officinalis

The bioactive components in the methanol extract from L. pubescens (LPME) stem bark evaluated by GC–MS are shown in Table 1 and Figure S5. Some compounds detected possess biological activities. The 4-Ethoxy-2-(methylamino)tropone (3.18% of the total peak area) has tropone derivatives that are active against bacteria, fungi, insects, and tumors. They also inhibit polyphenol oxidase activity [27,28]. The 6-phenanthidinol, 7,9-dimethyl-derivative phenanthidone acts as a mutagen and immunosuppressive agent [29]. The alkaloid 2(1H)-quinolinone, 3-hydroxy-4-(3-hydroxyphenyl)-(11.95%), has an anti-inflammatory effect on LPS-stimulated cells, resulting from the inhibition of the nuclear factor-kappa B (NF-κB) pathway [30]. The precursor of 9-(4-dimethylaminophenyl)anthracene (2.01%), that is, anthracene that can be used to synthesize pyrimidine pyrazoline-anthracene derivatives, exhibiting anti-cancer activity [31]. Ocatonoic acid (2.33%) has a potentiating effect on insulin secretion as observed in vivo [32]. Methyl nonanoate is a plant metabolite that is active against fungi and nematodes [33]. The 2,4-Di-tert-butylphenol was found in a considerable amount (12.24%), exhibiting auto-toxin ability and antioxidant activity [34]. Dodecanoic acid, methyl ester (2.57% of the total peak area) has a role as a metabolite [35]. Diethyl phthalate is a tetragonic agent, neurotoxin, endocrine disrupter, and hazardous substance to the environment [36]. Norepinephrine, ^®^-, 4TMS derivative contains the norepinephrine moiety, which is used in controlling blood pressure [37]. The GC–MS profile of the methanol extract also contains some fatty acid esters.

### 3.3. In Vitro Cytotoxicity

**Figure 2:**
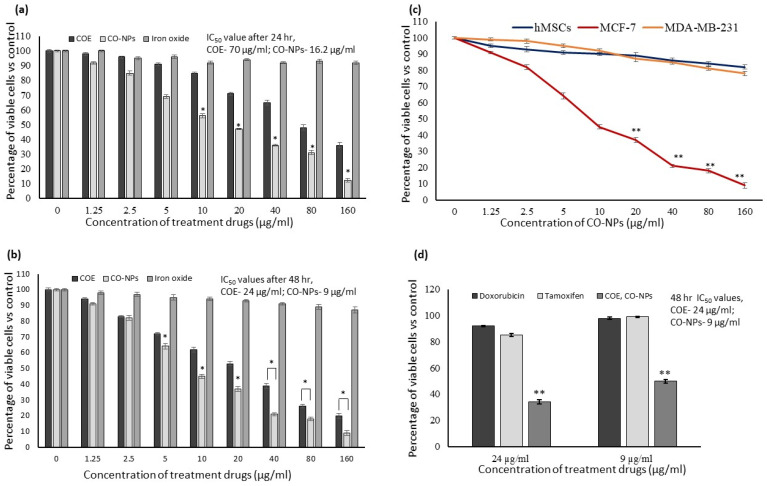
In vitro cytotoxicity of *Cinchona officinalis* extract (COE), iron oxide, and *Cinchona officinalis* extract-loaded iron oxide nanoparticles (CO-NPs) on MCF-7 breast cancer cells after 24 h (**a**) and 48 h (**b**). (**c**) shows the comparative cell growth inhibitory effect of CO-NPs using hMSCs, MCF-7, and MDA-MB-231 cells after 48 h. (**d**) shows the cell growth inhibitory effect of COE, CO-NPs vs doxorubicin and tamoxifen in MCF-7 cells after 48 h. The data are presented as means ± SD (*n* = 6). Values having an asterisk (*) or (**) are significantly different, compared with untreated cells (*p* ≤ 0.01 & *p* ≤ 0.05, respectively).

In vitro cytotoxicity assay confirmed that COE and CO-NPs significantly suppressed the MCF-7 cells’ growth within 24 h and increased in 48 h when compared to iron oxide or untreated control (Figure 2a,b). The IC_50_ doses of COE and CO-NPs were 24 µg/mL and 9 µg/mL after 48 h (Figure 2b). Only the MCF-7 cells’ growth was significantly (*p* ≤ 0.05) inhibited by CO-NPs treatment (91%); neither hMSCs (18%) nor MDA-MB-231 (22%) cells were significantly (*p* ≤ 0.05) impaired (Figure 2c). The IC_50_ doses of COE (24 µg/mL) and CO-NPs (9 µg/mL) were applied to doxorubicin and tamoxifen. The results showed that 24 µg/mL of doxorubicin inhibited only 8%, and tamoxifen inhibited 15% of MCF-7 cells. CO-NPs (9 g/mL) inhibited only 2% after doxorubicin treatment and 1% after tamoxifen treatment for 48 h (Figure 2d).

### 3.4. Cell and Nuclear Morphology

**Figure 3:**
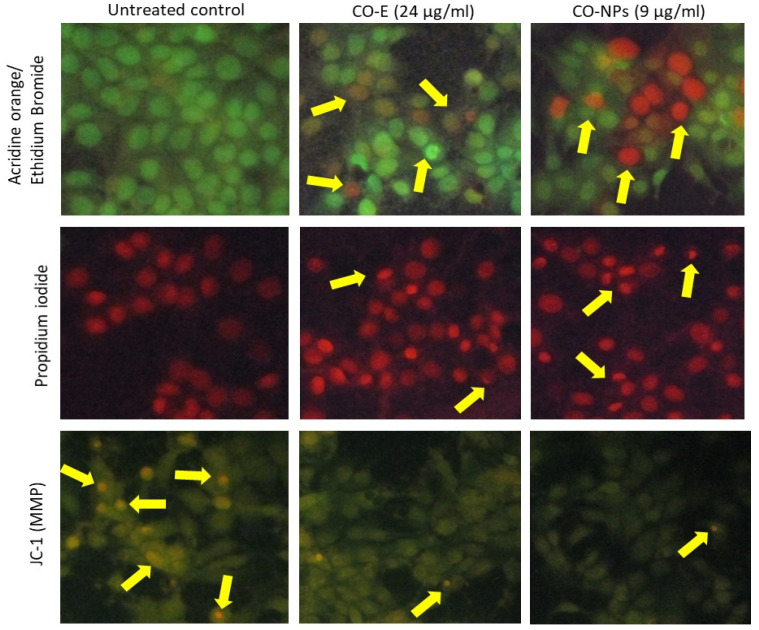
AO/ErBr staining, propidium iodide staining, and JC-1 staining images (200×) of untreated cells, and MCF-7 cells treated with *Cinchona officinalis* extract (COE) and *Cinchona officinalis* extract-loaded iron oxide nanoparticles (CO-NPs) for 48 h. IC_50_ doses are 24 µg/mL and 9 µg/mL for COE and CO-NPs, respectively.

Cellular and nuclear morphological variations were observed in IC_50_ doses of COE (24 µg/mL) and CO-NPs (9 µg/mL)-treated MCF-7 cells after 48 h (Figure 3). In comparison to COE treatment, PI staining revealed irregular shaped nuclei, indicating nuclear damage and apoptosis in MCF-7 cells after CO-NPs treatment. After being exposed to COE (24 g/mL) and CO-NPs (9 g/mL) for 48 h, AO/ErBr staining images revealed that some MCF-7 cells had dark green color, while others had light green, orange, and red colors, indicating the presence of pre-apoptotic, early apoptotic, late apoptotic, and necrotic cells, respectively (Figure 2b). The highest number of late apoptotic cells was observed at the 9 µg/mL dose of CO-NPs when compared to COE. Manual counting of apoptotic and necrotic cells within an average of 300 cells found that 83% in apoptotic and 6% were found with necrotic morphology.

### 3.5. JC-1 Staining to Determine the Mitochondrial Membrane Potential (Δψm)

Images of untreated MCF-7 cells (control), and the MCF-7 cells treated with 24 µg/mL of COE, and 9 µg/mL of CO-NPs after 48 h are shown in Figure 3. The JC-1 staining images displayed red confirmed the J-aggregates and green with JC-1 monomeric forms signals, corresponding to healthy intact membrane potential (polarized) and membrane potential lost or damaged (depolarized), respectively. In untreated cancer cells, a higher number of J-aggregates were found, representing the intact mitochondria and healthy cells. We discovered that treating MCF-7 breast cancer cells with 24 µg/mL of COE or 9 µg/mL of CO-NPs for 48 h resulted in lower J-aggregates, indicating the loss of mitochondrial membrane potential or metabolically inefficient cells. As can be seen, Δψm was significantly lower in MCF-7 cells treated with CO-NPs than COE.

### 3.6. Expressions Levels of Oxido-Redox and Tumor Suppressor Genes

**Figure 4:**
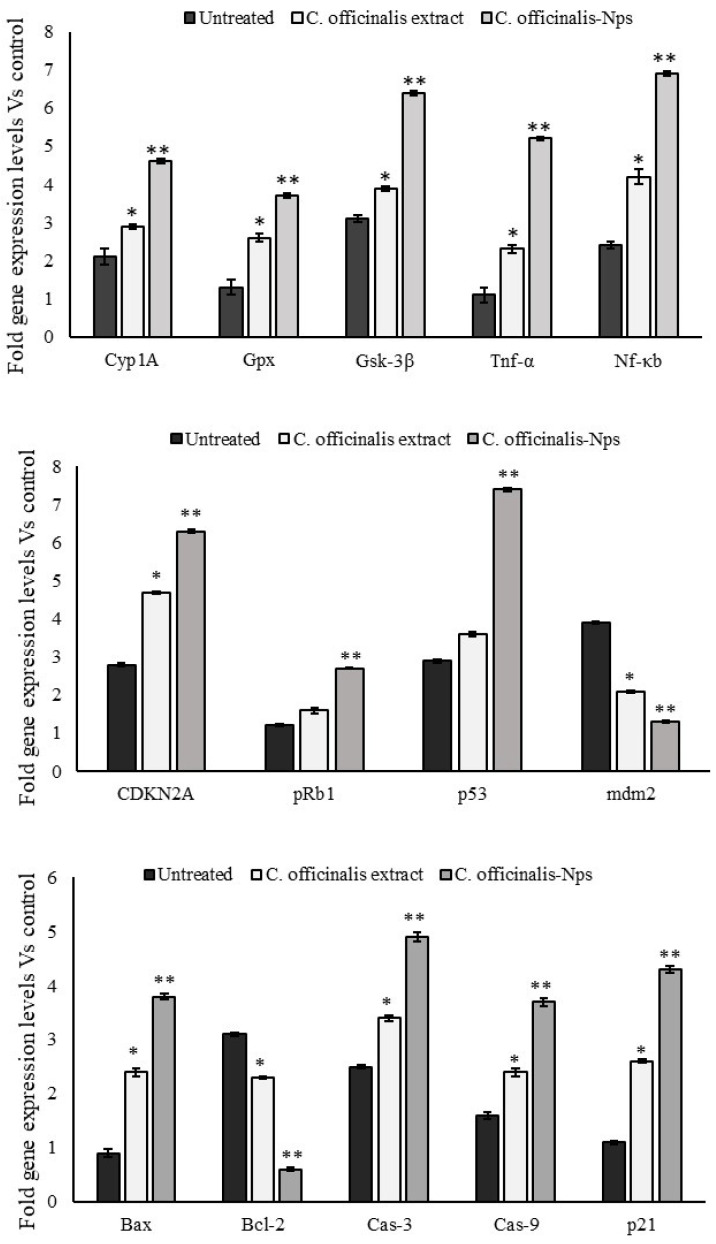
Effect of *Cinchona officinalis* extract (COE) and *Cinchona officinalis* extract-loaded iron oxide nanoparticles (CO-NPs) on oxidative stress, tumor suppressor, and expression levels of apoptotic genes in MCF-7 cells after 48 h treatment. The data are presented as means ± SD (*n* = 6). Values having an asterisk (*) or (**) are significantly different, compared with untreated cells (*p* ≤ 0.01 & *p* ≤ 0.05, respectively).

The quantitative PCR assay was used to measure how the MCF-7 breast cancer cells’ growth was affected after COE (24 µg/mL) and CO-NPs (9 µg/mL) treatment for 48 h (Figure 4). The levels of expression of oxidoreductase mediators (CYP1A, GSK-3β, and GPx), inflammatory markers (TNF-α and Nf-κb), tumor suppressors (Cdkn2a, pRb1, p53, and mdm2), and apoptotic mediators (BAX, Bcl-2, caspase-3, caspase-9, and p21) were investigated. The mitochondrial oxidative capacity-related genes, CYP1A, GSK3β, and GPx were significantly increased in MCF-7 cells after CO-NPs treatment compared with cells after COE treatment or untreated cells. Tumor suppressors, such as Cdkn2a, pRb1, p53, and mdm2, Bcl-2 levels were increased 2-fold in CO-NPs treated cells. The mitochondria-stimulating apoptotic genes, such as Bax, caspase-3, and p21 (Cdkn1a), expression levels increased while the mdm2 expression level decreased significantly.

## 4. Discussion

Breast cancer affects women globally and accounts for 23% of cancer incidence worldwide. It is the second largest cause of death in wealthy countries [38]. The current treatments for breast cancer include treatment with chemicals, radiation, and hormones, as well as surgery. Numerous clinical and environmental applications, such as drug administration, diagnostics, imaging, sensing, gene transfer, artificial implants, tissue engineering, parasitology, and pest control, have the potential to be modernized by adopting nanotechnology techniques. Modern treatments can not yet cure cancer in its later metastatic stages. Therefore, for the prevention and treatment of cancer, various innovative chemopreventive methods using natural phytochemicals have recently been developed [39,40]. Finding a new chemopreventive technique is therefore of utmost importance. Numerous benefits of nanomedicine for the treatment of cancer include site-specific targeting, the controlled biodistribution of nanosystems, and avoiding biological constraints to retain the bioavailability and pharmacodynamic properties of medications. As a result, nanotechnology offers a potential substitute for current cancer diagnosis and therapy options. Gold, silver, titanium, magnetic metal nanoparticles, etc., are commonly used to cure cancer [41]. Numerous studies targeting cancer cells, especially breast cancer, have used iron oxide nanoparticles functionalized with bioactive substances from different biological sources or drugs [40,42,43]. In this study, we successfully fabricated iron oxide nanoparticles (CO-NPs) using ferric chloride and the stem bark methanol extract of *C. officinalis* as a functionalizing agent. We proved the green synthesis, functionalization, particle size, and distribution of CO-NPs by various characterization methods, including UV-vis, XRD, TEM, and FT-IR. By using biological sources as the capping and reducing agents during the green production of iron oxide nanoparticles, biocompatible, environmentally friendly, and economically viable nanoparticles can be created [43,44,45,46]. Moreover, compared to other synthetic medicinal compounds, plant-based natural compounds are safe and simple to metabolize. It is believed that plant-based substances contribute to one-fourth of all pharmaceuticals used in industrialized nations [39]. In addition to tannins, quinine, quinidine, cinchonidine, and cinchonine are among the alkaloids found in the stem bark of *C. officinalis*. Several alkaloids and phenolics can suppress oxidative stress and inflammation and are active against tumors, microbes, and parasites [47]. The GC–MS profile of the methanol extract of *C. officinalis* stem bark demonstrated many bioactive compounds. For example, 2,4-Di-tert-butylphenol, (12.24%), exhibits auto-toxin ability and antioxidant activity [34]. The alkaloid 2 (1H)-quinolinone, 3-hydroxy-4-(3-hydroxyphenyl)-(11.95%), has an anti-inflammatory effect on LPS-stimulated cells, resulting from the inhibition of the NF-κB pathway [30]. Tropone derivatives of 4-Ethoxy-2-(methylamino)tropone (3.18%) are active against bacteria, fungi, insects, and tumors [27,28].

Several studies have examined the effectiveness of functionalized iron oxide nanoparticles in the treatment of cancer. It was found that they can induce apoptosis in cancer cells, including MCF-7 cells. An elevated proportion of MCF-7 cell apoptosis was noticed after treatment with functionalized iron oxide nanoparticles [40,41,42,43,48,49]. In this study, the cytotoxic effects of COE, iron oxide, and CO-PNs were investigated against hMSCs, MDA-MB-231, and MCF-7. However, the MTT cytotoxicity analysis proved that the functionalization of iron oxide nanoparticles by *C. officinalis* stem bark extract (CO-NPs) significantly reduced the MCF-7 cell viability (91%) after 48-h treatment, compared with COE, with an IC_50_ of 9 µg/mL, versus 24 µg/mL for COE (Section 3.3). Interestingly, the cytotoxicity effect of CO-NPs surpasses that of doxorubicin and tamoxifen anticancer drugs. The cytotoxicity analysis revealed that CO-NPs had no discernible impact on either hMSCs (18%) or MDA-MB-231 (22%).

A tumor-suppressive process that is engaged in response to stress signals causes programmed cell death, i.e., apoptosis. When death-signaling pathways are activated, susceptible target cells may experience an increase in death receptors, DNA destruction, mitochondrial malfunction, caspase production, a deficiency of growth factors, ER stress, and ROS damage. Apoptosis, which is characterized by cellular, morphological, and metabolic alterations, can be brought on by cytotoxic substances [50,51]. It uses two important routes; the extrinsic or cytoplasmic pathway is induced by the Fas death receptor signal [42]. The mitochondrial (intrinsic) pathway is the other apoptosis route, and when it is triggered, the mitochondria discharge the cytochrome c enzyme and switch on the death signal [52]. The activation of caspases, which are essential for apoptosis, necrosis, and inflammation, is the fundamental element of the death signal [42].

In the present study, cellular and nuclear morphological damage in MCF-7 cells were noticed after COE (IC_50_ = 24 µg/mL) and CO-NPs (IC_50_ = 9 µg/mL) treatments for 48 h. After CO-NPs treatment, PI staining images showed more MCF-7 cells with deformed, irregular-shaped nuclei when compared with COE (24 µg/mL), demonstrating nuclear damage and apoptosis. The results of AO/ErBr staining confirmed the occurrence of an array of apoptotic cells, classified as pre-apoptotic, early, and late apoptotic cells, as well as few necrotic cells, after COE and CO-NPs treatments. We observed a higher number of late apoptotic cells in CO-NPs than in COE, with 83% apoptotic cells and 6% necrotic cells. The nuclear damage after CO-PNs treatment could be stemming from the internalization of the nanoparticles in breast cancer cells. Numerous investigations have verified the internalization of the nanoparticles in treated cancer cells. For instance, Kavithaa et al. [42] discovered that MDA-MB-231 contained some baicalein-loaded iron nanoconjugates in the cytoplasmic vacuolar region and others in the mitochondrial region. Additionally, within 30 min, MCF-7 cells’ cytoplasm and vesicles accumulated superparamagnetic iron oxide nanoparticles loaded with dimercaptosuccinic acid, and after 6-h treatment, they accumulated towards the nuclei. Similar to this, glucose-loaded iron oxide nanoparticles were found in pancreatic adenocarcinoma cell vesicles and the nuclei.

Our results of JC-1 staining assured that untreated MCF-7 cells had a healthy mitochondrial membrane potential, while MCF-7 cells after 48 h of incubation following CO-NPs treatment had a marked lower mitochondrial membrane potential. The induction of intrinsic apoptosis, which is thought to be essential to the apoptotic pathway, is dependent on mitochondrial membrane dysfunction [42]. Biosynthesized iron oxide nanoparticles were reported to exert cytotoxicity on AMJ-13, MCF-7 [43,53], and MDA-MB-231 breast cancer cells [43]. Other cancer cell types such as lung adenocarcinoma A549 [54], hepatocellular carcinoma cell line HepG2 [55], and hepatoma cell line BEL-7402 [56] also showed a notable reduction in mitochondrial membrane potential after exposure to functionalized iron oxide nanoparticles.

Accordingly, it was clear that CO-NPs loaded with *C. officinalis* stem bark extract could enhance apoptosis in MCF-7 mitochondria, leading to intrinsic pathway activation. This was confirmed by examining the expression of specific tumor suppressors (Cdkn2a, pRb1, p53, and mdm2), oxidoreductase mediators (CYP1A, GSK-3β, and GPx), inflammatory markers (TNF-αand Nf-κb), and apoptotic genes (BAX, Bcl-2, Caspase-3, Caspase-9, and p21). Therefore, during a 48-h incubation period, we measured the toxicity of COE and CO-NPs on MCF-7 cells using the quantitative PCR technique. We discovered that CO-PNs significantly elevated the expression of the oxidative stress genes cytochrome P4501A (CYP1A), glycogen synthesis kinase-3beta (GSK-3 β), and glutathione peroxidase (GPx), as well as the cytokines tumor necrosis factor-alpha (TNF-α) and nuclear factor-kappa B (Nf-κb) in comparison to COE and untreated cells. This indicates the occurrence of oxidative stress and, consequently, DNA damage because of increased reactive oxygen species (ROS) that initiate the gene expressions related to the mitochondrial and extra-mitochondrial apoptotic pathways [57,58]. The overexpression of CYP1A can release ROS that leads to the augmentation of ROS over antioxidants, eventually causing inflammation and toxicity [59].

CO-NPs also significantly up-regulated mRNA tumor suppressor levels of p53, Cdkn2a (cyclin-dependent kinase inhibitor 2A) and pRb1 (retinoblastoma protein 1), and downregulated mdm2 (murine double minute 2) expression. The levels of apoptosis-induction genes, such as caspase-3, caspase-9, Bax, and Cdkn1a (cyclin-dependent kinase inhibitor 1A; p21), were also significantly higher in MCF-7 cells treated with CO-PNs than in COE and control cells. By contrast, the level of B-cell lymphoma 2 (Bcl-2) decreased significantly. The expressions of oxidoreductase genes (CYP1A, GSK-3, and GPx), as well as pro-inflammatory cytokines (TNF-α and Nf-κb), were increased in MCF-7 cells after 48 hr of CO-PNs treatment, demonstrating that cellular oxidative stress promotes apoptosis. Organ malfunction results from the activation of several intracellular signaling pathways by oxidative stress, which causes apoptosis or cell overgrowth [60]. The overexpression of the GPx gene serves to safeguard the free polyunsaturated fatty acids produced under the cellular stress environment and is a sign of the presence of oxidative stress. It performs as a component of a multi-component antioxidant defense system that, in the presence of reduced glutathione (GSH), catalyzes the conversion of H_2_O_2_ or organic peroxide (ROOH) to water or alcohol [61,62]. Oxidative stress also increases Nf-κB, which regulates the immune system’s response to infection, the development of cancer, and the occurrence of inflammatory and autoimmune diseases. The major transcription factor “rapid-acting” NF-B is the first to react to damaging cellular stimuli. Oxidative stress is essential for the development of CKD and is associated with endothelial dysfunction [62,63,64]. As can be seen, TNF-α and Nf-κB levels were significantly increased in MCF-7 after treatment with CO-PNs. TNF-α is believed to stimulate the development of breast cancer by upregulating the expression of the transcriptional co-activator TAZ [65]. Nevertheless, our results demonstrated that the high levels of pro-inflammatory cytokines did not induce the proliferation of MCF-7 cells after CO-NPs treatment. This could be due to TNF-α’s ability to synergistically act with some bioactive compounds loaded on the iron oxide nanoparticle to cause cancer cells to undergo apoptosis. In this regard, TNF-α is found to increase the cytotoxicity of chemotherapy drugs and radiation therapy against breast cancer cells both in vitro and in vivo [66]. The NF-κB pathway is activated by TNF-α, causing the expression of key regulators involved in the G1S phase transition to increase. Consequently, TNF-α shifts cancer cells from the dormant phase, G0/G1, to the more susceptible proliferative phase. TNF-α treatment can increase DNA damage after Cs^137^ irradiation and increase G2/M and S-phase cell cycle arrest caused by docetaxel and cisplatin drugs, respectively. TNF-α and 5-FU together can induce a significant sub-G0/G1 peak, implying that TNF-α-induced 5-FU sensitization may also involve the induction of necrosis or apoptosis. An increase in the expression of NF-κB has been noticed in breast cancer cells, MCF-10F, and MDA-MB-231, after Doxorubicin drug treatment [50]. Moreover, GSK-3β controls several signaling pathways that regulate a variety of biological activities, such as cell division, proliferation, apoptosis, and metabolism. It modulates the production of the inhibitor-of-apoptosis protein (IAP) survivin, hence controlling apoptosis in human lung cancer cells [67]. It participates in the cellular reaction to oxidative stress by regulating the nuclear factor erythroid 2-related factor 2 (Nrf2) [68].

As mentioned above, Cdkn2A, pRb1, and p53, defined as tumor suppressor genes, increased in MCF-7 after CO-PNs treatment, while the mdm2 level decreased. Apoptotic genes like caspase-3, caspase-9, p21, Bax (over-regulated), and Bcl-2 (downregulated) were changed in response to CO-NPs treatment. In addition to being involved in cell cycle development, repair of damaged DNA, and apoptosis, p53 suppresses cellular stress-induced carcinogenesis. Pro-apoptotic genes associated with intrinsic and extrinsic pathways are transcriptionally regulated by P53 [69]. In our study, P53 was upregulated, probably due to the downregulation of mdm2, increasing the p21 expression in cells with damaged DNA [50]. Further, Bax gene expression was elevated in the MCF-7 cell line after CO-NPs treatment and Bcl2 was downregulated, suggesting that CO-PNs influenced the expression of members of the Bcl-2 family [50]. Cancer cells frequently have lower levels of the Bcl-2 family proteins, which control the apoptotic threshold, whereas Bax levels are higher [39,70]. In breast cancer, overexpressed mutant p53 is responsible for the decrease in expression levels of Bcl-2 [71]. Bcl-2 controls apoptosis by being involved in the shift from the G0/G1 to the S phase of the cell cycle, which is responsible for controlling the cell cycle and proliferation [72]. The pro-apoptotic gene Bax is upregulated when p53 is activated, in contrast to Bcl-2. Fas and the intrinsic mitochondrial pathway, which activates caspase-8 and caspase-9 [73], and Bax expression, which causes the release of cytochrome c and mitochondrial dysfunction, are all prompted by P53-mediated apoptosis. Bax is an essential pro-apoptotic protein. It promotes apoptosis by interacting with Bcl-2 to restrict Bcl-2 function. Bax can also speed up the opening of the mitochondrial voltage-dependent anion channel, which causes the mitochondrial membrane potential to collapse, cytochrome c to be released, and caspase-3 to be activated before apoptosis is initiated [74].

Endometrial cells are however more susceptible to apoptosis when the Bcl-2/Bax ratio is low, and an increased Bcl-2/Bax ratio within a cell is a key indicator of apoptosis. According to certain studies, disruption of the p53/Bcl-2/Bax apoptotic signaling pathway plays a role in the initiation and growth of malignancies [75]. The one most extensively researched is the effector caspase 3. It is vital for the death receptor and the mitochondrial routes, which are initiated by caspase-8 and caspase-9, respectively. Additionally, multiple investigations have revealed that the induction of apoptosis in response to chemotherapy drugs like doxorubicin requires caspase-3 activation [76]. Numerous cellular substrates and DNA repair enzymes are destroyed by caspase-3, whereas it stimulates a DNA-fracturing endonuclease as part of apoptosis [77]. The caspases implicated in apoptosis are classified as initiator and effector caspases [78]. One important protease that is activated in the initial stages of apoptosis is caspase-3. In the cytoplasm or nucleus, caspase-3 enhances other caspases as well as other relevant molecules [79,80]. As a result, caspase-3 is a potential marker to determine whether a breast cancer patient will respond to or be resistant to chemotherapeutic drugs [76]. Cytochrome c might be released from the mitochondria during cell death, which would then cause the caspases to be activated and cause apoptosis. In response to cytochrome c release from the mitochondria, a particular cascade of activation between caspase-9 and -3 occurs; as a result, effector caspases like caspase-3, -6, and -7 are cleaved [81,82]. Accordingly, the effector caspases cleave their intended proteins, and the cell eventually dies in a controlled, programmed manner. The essential players in the apoptosis route are caspase-3 and caspase-9, whose actions impact both the manner of cell death and the apoptosis process [51]. According to Yang et al. [83], and Taherzadeh-Soureshjani and Chehelgerdi [38], caspase-3 may then be able to cleave a variety of proteins like a DNA repair enzyme, Poly (ADP-ribose) polymerase (PARP). Additionally, pro-apoptosis is indicated by the significant increase in pRb1 and Cdkn2a gene expression in MCF-7 following treatment with CO-PNs. A transcriptional repressor, pRb1 prevents proliferation at the G1/S transition by binding to and deactivating E2F, a transcription-factor family member. It also regulates cellular differentiation. The essential tumor suppressor Cdkn2a gene (Ink4a) is the main regulator of pRb1 [84].

The results of the present study implied that CO-PNs generated oxidative stress in MCF-7 cells by upregulating the expression of the pro-apoptotic gene Bax and down-regulating of Bcl-2 by tumor suppression and pro-apoptosis genes. Similar changes in pro-apoptotic genes and tumor suppression genes in breast cancer cells MDA-MB-231 [40,42,85] and MCF-7 [45,53] as a result of functionalized metal nanoparticle treatment have been reported earlier. Since *C. officinalis* stem bark extract is rich in bioactive compounds like alkaloids, phenolics, flavonoids, etc., it was reported to have an apoptotic impact on cancer cells [40,86,87]. The higher cytotoxicity of the synthesized iron oxide nanoparticles against MCF-7 cells compared with the iron oxide and COE alone suggests that the synthesized CO-NPs have been adequately loaded with *C. officinalis* stem bark extract. Finally, the biosynthesis of metal oxide nanoparticles functionalized with stem bark extract can assist in the improvement of the nanoparticle cytotoxicity against MCF-7 cell lines, which in turn stimulates the gene expression pathways, resulting in oxidative stress, and consequently apoptosis.

## 5. Conclusions

This study demonstrated that successfully functionalized iron oxide nanoparticles with bioactive compounds of *C. officinalis* stem bark extract (CO-PNs) induced apoptosis in the breast cancer cell MCF-7. The flow cytometric analysis revealed that the CO-NPs increased the extent of apoptosis, cellular oxidative stress, and DNA damage in MCF-7 cells. Simultaneously, apoptotic and anti-apoptotic gene expression patterns showed that CO-NPs enhanced apoptosis by activating apoptotic genes, such as Bax, caspase-3, caspase-9, and p21, and inhibiting the expression of anti-apoptotic genes, such as Bcl-2. Our findings allow us to speculate that using CO-NPs against MCF-7 cells could pave the way for the future use of iron nanoparticles in cancer drug delivery systems.

## Figures and Tables

**Table 1:** GC–MS profile of bioactive components in *C. officinalis* stem bark methanol extract.

No.	RT (min)	Peak Area (%)	Compound Name	Molecular Formula	Molecular Weight (g/mol)	Compound Nature	Bioactivity
1	5.36	3.18	4-Ethoxy-2-(methylamino) tropone	C_10_H_13_NO_2_	179.22	Cyclic aliphatic ketone	Tropone derivatives function as anti-ischemic, insecticidal, bacterial, fungal, and anti-tumor agents. Additionally, they can deactivate polyphenol oxidase and chelate iron [27,28].
2	6.72	3.01	6-phenanthidinol, 7,9-dimethyl-	C_15_H_13_NO	223.27	phenanthidine	A mutagen and immunosuppressive agent [29].
3	9.77	11.95	2(1H)-Quinolinone, 3-hydroxy-4-(3-hydroxyphenyl)-(Viridicatol)	C_15_H_11_NO_3_	253.25	Alkaloid	Viridicatol acts as an anti-inflammatory agent [30].
5	10.42	2.01	9-(4-Dimethylaminophenyl)anthracene	C_22_H_19_N	297.4	Cyclic hydrocarbon	Pyrimidine pyrazoline-anthracene derivatives are active against normal fibroblast cells and hepatocellular carcinoma cells [31].
6	12.05	2.33	Octanoic acid, methyl ester (Methyl octanoate)	C_9_H_18_O_2_	158.24	Fatty acid ester	It is a metabolite. It has a potentiating effect on insulin secretion [32].
7	14.49	2.46	Nonanoic acid, methyl ester (Methyl pelarigonate)	C_10_H_20_O_2_	172.26	Fatty acid ester	It is an epitope, antifungal agent, and antinematodal drug, as well as a plant metabolite [33].
8	20.76	12.24	2,4-Di-tert-butylphenol	C_14_H_22_O	206.32	Alkylbenzene and a member of phenols	An auto-toxin, antioxidant, bacterial metabolite, and marine metabolite [34].
9	21.13	2.57	Dodecanoic acid, methyl ester	C_14_H_28_O_2_	228.37	Fatty acid ester	It has a role as a metabolite [35].
10	22.41	2.63	Diethyl Phthalate	C_12_H_14_O_4_	222.24	Phthalate ester	A tetragonic agent, neurotoxin, endocrine disrupter, and a hazardous substance to the environment [36].
11	23.17	9.06	Norepinephrine, (R)-, 4TMS derivative	C_20_H_43_NO_3_Si_4_	457.9	Catecholamine	The norepinephrine moiety is used in the control of blood pressure [37].
12	26.14	6.82	Methyl 9-methyltetradecanoate	C_16_H_32_O_2_	256.42	Fatty acid ester	Not reported
13	26.26	9.90	Heptacos-1-ene	C_27_H_54_	378.7	alkene	Not reported
14	30.12	6.10	Heptadcanoic acid, methyl ester	C_18_H_36_O_2_	284.5	Fatty acid ester	Not reported
15	31.28	1.92	9-Octadecenoic acid (Z)-, methyl ester (Methyl Oleate)	C_19_H_36_O_2_	296.50	Fatty acid ester	Not reported
16	31.37	5.52	Cyclopropaneoctanoic acid, 2-hexyl-, methyl ester	C_18_H_34_O_2_	282.5	Fatty acid ester	Not reported
17	34.62	2.72	Eicosanoic acid, methyl ester (Methyl arachidate)	C_21_H_42_O_2_	326.6	Fatty acid ester	Not reported

## Data Availability

The datasets used and analyzed during the current study are available from the corresponding author upon reasonable request.

## Supplementary Materials

The following supporting information can be downloaded at: https://www.mdpi.com/article/10.3390/nano12193393/s1, Figure S1: *C. officinalis* stem bark methanol extract (A), iron oxide (B) and iron oxide nanoparticle solutions ((C) 50 mg extract + 0.1 M FeCl_3_; (D) 100 mg extract + 0.1 M FeCl_3_); Figure S2: UV-vis spectra. Iron oxide NPs prepared by reacting 50 mg and 100 mg of *C. officinalis* stem bark methanol extract with 0.05 M (A) and 0.1 M (B) FeCl_3_ solutions; Figure S3: XRD spectra. Iron oxide (a). Iron oxide NPs prepared by reacting 0.05 M and 0.1 M FeCl_3_ with 50 mg (b,d) and 100 mg (c,e) of *C. officinalis* stem bark methanol extract, respectively; Figure S4: FT-IR spectra of *C. officinals* extract, iron oxide, and 50 mg and 100 mg NPs prepared using FeCl_3_ at concentrations of 0.05 M (a) and 0.1 M (b); Figure S5: GC-MS chromatograms of the methanol extract components of *C. officinals* stem bark.

## References

[1] World Health Organization (2021). Breast Cancer.

[2] Alqahtani W.S., Almufareh N.A., Domiaty D.M., Albasher G., Alduwish M.A., Alkhalaf H., Almuzzaini B., AL-marshidy S.S., Alfraihi R., Elasbali A.M. (2020). Epidemiology of cancer in Saudi Arabia thu 2010–2019: A systematic review with constrained meta-analysis. AIMS Public Health.

[3] Akram M., Iqbal M., Daniyal M., Khan A.U. (2017). Awareness and current knowledge of breast cancer. Biol. Res..

[4] Comsa Ş., Cimpean A.M., Raica M. (2015). The Story of MCF-7 Breast Cancer Cell Line: 40 years of Experience in Research. Anticancer Res..

[5] Bukhari A., Ijaz I., Gilani E., Nazir A., Zain H., Saeed R., Alarfaji S.S., Hussain S., Aftab R., Naseer Y. (2021). Green synthesis of metal and metal oxide nanoparticles using different plants’ parts for antimicrobial activity and anticancer activity: A review article. Coatings.

[6] Evans E.R., Bugga P., Asthana V., Drezek R. (2018). Metallic nanoparticles for cancer immunotherapy. Mater. Today.

[7] Siddiqi K.S., ur Rahman A., Tajuddin, Husen A. (2016). Biogenic fabrication of iron/iron oxide nanoparticles and their application. Nanoscale Res. Lett..

[8] Kuppusamy P., Yusoff M.M., Maniam G.P., Govindan N. (2016). Biosynthesis of metallic nanoparticles using plant derivatives and their new avenues in pharmacological applications—An updated report. Saudi Pharm. J..

[9] Raza M.A., Rehman F., ur Anwar S., Zaha A., Rehman A., Rashid E., Kalsoom M., Ilahi H. (2021). The Medicinal Aromatic Activities of cinchona: A Review. Asian J. Adv. Res..

[10] Mosmann T. (1983). Rapid colorimetric assay for cellular growth and survival: Application to proliferation and cytotoxicity assays. J. Immunol. Methods.

[11] Leite M., Quinta-Costa M., Leite P.S., Guimaraes J.E. (1999). Critical evaluation of techniques to detect and measure cell death–study in a model of UV radiation of the leukaemic cell line HL60. Anal. Cell. Pathol..

[12] Yuan J.S., Reed A., Chen F., Stewart C.N. (2006). Statistical analysis of real–time PCR data. BMC Bioinform..

[13] Bhuiyan M.S., Miah M.Y., Paul S.C., Aka T.D., Saha O., Rahaman M.M., Sharif M.J.I., Habiba O., Ashaduzzaman M. (2020). Green synthesis of iron oxide nanoparticle using Carica papaya leaf extract: Application for photocatalytic degradation of remazol yellow RR dye and antibacterial activity. Heliyon.

[14] Guivar J.A.R., Martínez A.I., Ana Osorio Anaya A.O., Luis De Los Santos Valladares L.D.-L.S., Félix L.L., Dominguez A.B. (2014). Structural and magnetic properties of monophasic maghemite (*γ*-Fe_2_O_3_) nanocrystalline Powder. Adv. Nanoparticles.

[15] Liang C., Liu H., Zhou J., Peng X., Zhang H. (2015). One-step Synthesis of spherical 𝛾-Fe_2_O_3_ panopowders and the evaluation of their thotocatalytic activity for orange I degradation. J. Chem..

[16] Sahoo S.K., Agarwal k Singh A.K., Polke B.G., Raha K.C. (2010). Characterization of γ- and α-Fe_2_O_3_ nano powders synthesized by emulsion precipitation-calcination route and rheological behaviour of α-Fe_2_O_3_. Int. J. Eng. Sci. Technol..

[17] Islam M.S., Kurawaki J., Kusumoto Y., Abdulla-Al-Mamun M., Bin Mukhlish M.Z. (2012). Hydrothermal Novel Synthesis of Neck-structured Hyperthermia-suitable Magnetic (Fe3O4, γ-Fe_2_O_3_ and α-Fe_2_O_3_) Nanoparticles. J. Sci. Res..

[18] Valášková M., Tokarský J., Pavlovský J., Prostějovský T., Kočí K. (2019). α-Fe_2_O_3_ nanoparticles/vermiculite clay material: Structural, optical and photocatalytic properties. Materials.

[19] Alshammari G.M., Yagoub A.E.A., Subash-Babu P., Hassan A.B., Al-Nouri D.M., Mohammed M.A., Yahya M.A., Elsayim R. (2021). Inhibition of Lipid Accumulation and Adipokine Levels in Maturing Adipocytes by *Bauhinia rufescens* (Lam.) Stem Bark Extract Loaded Titanium Oxide Nanoparticles. Molecules.

[20] Mohammed A.E., Al-Qahtani A., Al-Mutairi A., Al-Shamri B., Aabed K. (2018). Antibacterial and cytotoxic potential of biosyn-thesized silver nanoparticles by some plant extracts. Nanomaterials.

[21] Hariharan D., Srinivasan K., Nehu L. (2017). Synthesis and characterization of TiO_2_ nanoparticles using Cynodon dactylon leaf extract for antibacterial and anticancer (A549 Cell Lines) Activity. J. Nanomed. Res..

[22] Aisida S.O., Akpa P.A., Ahmad I., Ting-kai Zhao T.K., Maaza M., Ezem F.I. (2020). Bio-inspired encapsulation and functionalization of iron oxide nanoparticles for biomedical applications. Eur. Polym. J..

[23] Manrique G.D., Lajolo F.M. (2002). FT-IR spectroscopy as a tool for measuring degree of methyl esterification in pectins isolated from ripening papaya fruit. Postharvest Biol. Technol..

[24] Qasim S., Zafar A., Saif M.S., Ali Z., Nazar M., Waqas M., Haq A.U., Tariq T., Hassan S.G., Iqbal F. (2020). Green synthesis of iron oxide nanorods using Withania coagulans extract improved photocatalytic degradation and antimicrobial activity. J. Photochem. Photobiol. B Biol..

[25] Bala N., Saha S., Maiti M., Sarkar M., Das S., Nandi P., Basu R. (2016). Riboflavin conjugated temperature variant ZnO nanoparticles with potential medicinal application in jaundice. RSC Adv..

[26] English N.J., Rahman M., Wadnerkar N., MacElroy J.M.D. (2014). Photo-active and dynamical properties of hematite (Fe_2_O_3_)–water interfaces: An experimental and theoretical study. Phys. Chem. Chem. Phys..

[27] Saniewski M., Horbowicz M., Kanlayanarat S. (2014). The Biological Activities of Troponoids and Their Use in Agriculture A Review. J. Hortic. Res..

[28] Cao F., Orth C., Donlin M.J., Adegboyega P., Meyers M., Murelli R.P., Elagawany M., Elgendy B., Tavis J.E. (2018). Synthesis and Evaluation of Troponoids as a New Class of Antibiotics. ACS Omega.

[29] ChEBI Phenanthidone. https://www.ebi.ac.uk/chebi/searchId.do?chebiId=CHEBI:75292..

[30] Insight Drugs Viridicatol. National Center for Advancing Translational Sciences. https://drugs.ncats.io/drug/45P12JNE0L.

[31] Ahmed N.M., Youns M., Soltan M.K., Said A.M. (2019). Design, synthesis, molecular modelling, and biological evaluation of novel substituted pyrimidine derivatives as potential anticancer agents for hepatocellular carcinoma. J. Enzym. Inhib. Med. Chem..

[32] Zhang T., Chen P., Stanley C.A., Hoshi T., Li C. (2019). Mechanisms of octanoic acid potentiation of insulin secretion in isolated islets. Islets.

[33] ChEBI Methyl Nonanoate. https://www.ebi.ac.uk/chebi/searchId.do?chebiId=CHEBI:44499.

[34] ChEBI 2,4-Di-Tert-Butylphenol. https://www.ebi.ac.uk/chebi/searchId.do?chebiId=CHEBI:89188.

[35] ChEBI Methyl Dodecanoate. https://www.ebi.ac.uk/chebi/searchId.do?chebiId=CHEBI:87494.

[36] ChEBI Diethyl Phthalate. https://www.ebi.ac.uk/chebi/searchId.do?chebiId=CHEBI:34698.

[37] DrugBank Norepinephine, (R)-, 4TMS Derivative. https://go.drugbank.com/drugs/DB00368.

[38] Arshi A., Sharifi F.S., Ghahfarokhi M.K., Faghih Z., Doosti A., Ostovari S., Maymand E.M., Seno M.M.G. (2018). Expression Analysis of MALAT1, GAS5, SRA, and NEAT1 lncRNAs in Breast Cancer Tissues from Young Women and Women over 45 Years of Age. Mol. Ther.-Nucleic Acids.

[39] Taherzadeh-Soureshjani P., Chehelgerdi M. (2020). Algae-meditated route to cuprous oxide (Cu_2_O) nanoparticle: Differential expression profile of MALAT1 and GAS5 LncRNAs and cytotoxic effect in human breast cancer. Cancer Nanotechnol..

[40] Taherian A., Esfandiari N., Rouhani S. (2021). Breast cancer drug delivery by novel drug-loaded chitosan-coated magnetic nanoparticles. Cancer Nanotechnol..

[41] Siafaka P.I., Okur N.Ü., Karantas I.D., Okur M.E., Gündoğdu E.A. (2021). Current update on nanoplatforms as therapeutic and diagnostic tools: A review for the materials used as nanotheranostics and imaging modalities. Asian J. Pharm. Health Sci..

[42] Kavithaa K., Sumathi S., Padma P.R. (2017). Intracellular Uptake of PEG-Funtionalized Baicalein Loaded Iron Oxide Nanoparticles Regulates Apoptotic Genes in Triple Negative Breast Cancer Cells: Mitochondrial Pathway Targeted Therapy for Breast Cancer. J. Clust. Sci..

[43] Thenmozhi T. (2020). Functionalization of iron oxide nanoparticles with clove extract to induce apoptosis in MCF-7 breast cancer cells. 3 Biotech.

[44] Benelli G. (2016). Green synthesized nanoparticles in the fight against mosquito-borne diseases and cancer—A brief review. Enzym. Microb. Technol..

[45] Ahamed M., Akhtar M.J., Alhadlaq H.A., Alshamsan A. (2016). Copper ferrite nanoparticle-induced cytotoxicity and oxidative stress in human breast cancer MCF-7 cells. Colloids Surf. B Biointerfaces.

[46] Benelli G., Lukehart C.M. (2017). Special Issue: Applications of Green-Synthesized Nanoparticles in Pharmacology, Parasitology and Entomology. J. Clust. Sci..

[47] Gurung P., De P. (2017). Spectrum of biological properties of cinchona alkaloids: A brief review. J. Pharmacogn. Photochem..

[48] Hosseinkazemi H., Samani S., O’Neill A., Soezi M., Moghoofei M., Azhdari M.H., Aavani F., Nazbar A., Keshel S.H., Doroudian M. (2022). Applications of Iron Oxide Nanoparticles against Breast Cancer. J. Nanomater..

[49] Alangari A., Alqahtani M.S., Mateen A., Kalam M.A., Alshememry A., Ali R., Kazi M., AlGhamdi K.M., Syed R. (2022). Iron Oxide Nanoparticles: Preparation, Characterization, and Assessment of Antimicrobial and Anticancer Activity. Adsorpt. Sci. Technol..

[50] Pilco-Ferreto N., Calaf G.M. (2016). Influence of doxorubicin on apoptosis and oxidative stress in breast cancer cell lines. Int. J. Oncol..

[51] Balachandran C., Sangeetha B., Duraipandiyan V., Raj M.K., Ignacimuthu S., Al-Dhabi N., Balakrishna K., Parthasarathy K., Arulmozhi N., Arasu M.V. (2014). A flavonoid isolated from Streptomyces sp. (ERINLG-4) induces apoptosis in human lung cancer A549 cells through p53 and cytochrome c release caspase dependant pathway. Chem. Interact..

[52] Hockenbery D.M., Nuñez G., Milliman C.L., Schreiber R.D., Korsmeyer S.J. (1990). Bcl-2 is an inner mitochondrial membrane protein that blocks programmed cell death. Nature.

[53] Sulaiman G.M., Tawfeeq A.T., Naji A.S. (2018). Biosynthesis, characterization of magnetic iron oxide nanoparticles and evaluations of the cytotoxicity and DNA damage of human breast carcinoma cell lines. Artif. Cells Nanomed. Biotechnol..

[54] Khan M.I., Mohammad A., Patil G., Naqvi S., Chauhan L., Ahmad I. (2012). Induction of ROS, mitochondrial damage and autophagy in lung epithelial cancer cells by iron oxide nanoparticles. Biomaterials.

[55] He C., Jiang S., Jin H., Chen S., Lin G., Yao H., Wang X., Mi P., Ji Z., Lin Y. (2016). Mitochondrial electron transport chain identified as a novel molecular target of SPIO nanoparticles mediated cancer-specific cytotoxicity. Biomaterials.

[56] Kai W., Xiaojun X., Ximing P., Zhenqing H., Qiqing Z. (2011). Cytotoxic effects and the mechanism of three types of magnetic nanoparticles on human hepatoma BEL-7402 cells. Nanoscale Res. Lett..

[57] Zhu M.-T., Wang B., Wang Y., Yuan L., Wang H.-J., Wang M., Ouyang H., Chai Z.-F., Feng W.-Y., Zhao Y.-L. (2011). Endothelial dysfunction and inflammation induced by iron oxide nanoparticle exposure: Risk factors for early atherosclerosis. Toxicol. Lett..

[58] Al-Saran N., Subash-Babu P., Al-Nouri D.M., Alfawaz H.A., Alshatwi A.A. (2016). Zinc enhances CDKN2A, pRb1 expression and regulates functional apoptosis via upregulation of p53 and p21 expression in human breast cancer MCF-7 cell. Environ. Toxicol. Pharmacol..

[59] Stading R., Chu C., Couroucli X., Lingappan K., Moorthy B. (2020). Molecular role of cytochrome P4501A enzymes in oxidative stress. Curr. Opin. Toxicol..

[60] Ogura S., Shimosawa T. (2014). Oxidative Stress and Organ Damages. Curr. Hypertens. Rep..

[61] Gathwala G., Aggarwal R. (2016). Selenium supplementation for the preterm Indian neonate. Indian J. Public Health.

[62] Tejchman K., Kotfis K., Sieńko J. (2021). Biomarkers and Mechanisms of Oxidative Stress—Last 20 Years of Research with an Emphasis on Kidney Damage and Renal Transplantation. Int. J. Mol. Sci..

[63] Prabhakar O. (2013). Cerebroprotective effect of resveratrol through antioxidant and anti-inflammatory effects in diabetic rats. Naunyn-Schmiedebergs Arch. Exp. Pathol. Pharmakol..

[64] Heissig B., Salama Y., Takahashi S., Osada T., Hattori K. (2020). The multifaceted role of plasminogen in inflammation. Cell Signal..

[65] Liu W., Lu X., Shi P., Yang G., Zhou Z., Li W., Mao X., Jiang D., Chen C. (2020). TNF-α increases breast cancer stem-like cells through up-regulating TAZ expression via the non-canonical NF-κB pathway. Sci. Rep..

[66] Wu X., Wu M.-Y., Jiang M., Zhi Q., Bian X., Xu M.-D., Gong F.-R., Hou J., Tao M., Shou L.-M. (2017). TNF-α sensitizes chemotherapy and radiotherapy against breast cancer cells. Cancer Cell Int..

[67] Li J., Xing M., Zhu M., Wang X., Wang M., Zhou S., Li N., Wu R., Zhou M. (2008). Glycogen synthase kinase 3β induces apoptosis in cancer cells through increase of survivin nuclear localization. Cancer Lett..

[68] Culbreth M., Aschner M. (2018). GSK-3β, a double-edged sword in Nrf2 regulation: Implications for neurological dysfunction and disease. F1000Research.

[69] Liu J., Zhang C., Feng Z. (2013). Tumor suppressor p53 and its gain-of-function mutants in cancer. Acta Biochim. Biophys. Sin..

[70] Delbridge A.R.D., Strasser A. (2015). The BCL-2 protein family, BH3-mimetics and cancer therapy. Cell Death Differ..

[71] Cho M.-Y., Park S.-Y., Park S., Lee Y.R., Kwak M.-K., Kim J.-A. (2012). Effects of geranyl-phloroacetophenone on the induction of apoptosis and chemosensitization of adriamycin-resistant MCF-7 human breast cancer cells. Arch. Pharmacal Res..

[72] Bonnefoy-Berard N., Aouacheria A., Verschelde C., Quemeneur L., Marçais A., Marvel J. (2004). Control of proliferation by Bcl-2 family members. Biochim. Biophys. Acta (BBA)—Mol. Cell Res..

[73] Liebermann D.A., Hoffman B., Vesely D. (2007). p53 Induced Growth Arrest versus Apoptosis and its Modulation by Survival Cytokines. Cell Cycle.

[74] Lee Y.K., Choi E.-J., Webster T.J., Kim S.-H., Khang D. (2014). Effect of the protein corona on nanoparticles for modulating cytotoxicity and immunotoxicity. Int. J. Nanomed..

[75] Argiris A., Cohen E., Karrison T., Esparaz B., Mauer A., Ansari R., Wong S., Lu Y., Pins M., Dancey J. (2006). A phase II trial of perifosine, an oral alkylphospholipid, in recurrent or metastatic head and neck cancer. Cancer Biol. Ther..

[76] O’Donovan N., Crown J., Stunell H., Hill A.D., McDermott E., O’Higgins N., Duffy M.J. (2003). Caspase 3 in breast cancer. Clin. Cancer Res..

[77] Stennicke H.R., Salvesen G.S. (1998). Properties of the caspases. Biochim. Biophys. Acta (BBA)—Protein Struct. Mol. Enzym..

[78] Mancini M., Nicholson D.W., Roy S., Thornberry N.A., Peterson E.P., Casciola-Rosen L.A., Rosen A. (1998). The Caspase-3 Precursor Has a Cytosolic and Mitochondrial Distribution: Implications for Apoptotic Signaling. J. Cell Biol..

[79] Brentnall M., Rodriguez-Menocal L., De Guevara R.L., Cepero E., Boise L.H. (2013). Caspase-9, caspase-3 and caspase-7 have distinct roles during intrinsic apoptosis. BMC Cell Biol..

[80] Walsh J.G., Cullen S.P., Sheridan C., Lüthi A.U., Gerner C., Martin S.J. (2008). Executioner caspase-3 and caspase-7 are functionally distinct proteases. Proc. Natl. Acad. Sci. USA.

[81] Shalini S., Dorstyn L., Dawar S., Kumar S. (2015). Old, new and emerging functions of caspases. Cell Death Differ..

[82] Porter A.G., Jänicke R.U. (1999). Emerging roles of caspase-3 in apoptosis. Cell Death Differ..

[83] Yang L.-L., Chang C.-C., Chen L.-G., Wang C.-C. (2003). Antitumor Principle Constituents of *Myrica rubra* Var. *acuminata*. J. Agric. Food Chem..

[84] Pomerantz J.H., Blau H.M. (2013). Tumor suppressors: Enhancers or suppressors of regeneration?. Development.

[85] ABU N., Akhtar M.N., Yeap S.K., Lim K.L., Ho W.Y., Zulfadli A.J., Omar A.R., Sulaiman M.R., Abdullah M.P., Alitheen N.B. (2014). Flavokawain A Induces Apoptosis in MCF-7 and MDA-MB231 and Inhibits the Metastatic Process In Vitro. PLoS ONE.

[86] Yallapu M.M., Othman S.F., Curtis E.T., Bauer N.A., Chauhan N., Kumar D., Jaggi M., Chauhan S.C. (2012). Curcumin-loaded magnetic nanoparticles for breast cancer therapeutics and imaging applications. Int. J. Nanomed..

[87] Krishnaveni M., Suresh K. (2015). Induction of apoptosis by quinine in human laryngeal carcinoma cell line (KB). Int. J. Curr. Res. Acad. Rev..

