# Supplementary material for: Cinchona officinalis Phytochemicals-Loaded Iron Oxide Nanoparticles Induce Cytotoxicity and Stimulate Apoptosis in MCF-7 Human Breast Cancer Cells
Source: Nanomaterials (Basel). 2022 Sep 28;12(19):3393. doi: 10.3390/nano12193393 (PMC9565860; doi:10.3390/nano12193393)
Supplement: Supplementary file 1 [file nanomaterials-12-03393-s001.zip › nanomaterials-1879723-supplementary.pdf]

nanomaterials

Supplementary materials

Cinchona officinalis Phytochemicals-Loaded Iron Oxide Nanoparticles Induce Cytotoxicity and Stimulate Apoptosis in MCF-7 Human Breast Cancer Cells

Laila Naif Al-Harbi *, Ghedier M. Al-Shammari *, Pandurangan Subash-Babu, Mohammed A. Mohammed, Roaa Ahmed Alkreadees and Abu ElGasim Ahmed Yagoub

Department of Food Science and Nutrition, College of Food Science and Agriculture, King Saud University, Riyadh 11451, Saudi Arabia
* Correspondence: lalharbi1@ksu.edu.sa (L.N.A.-H.); aghedeir@ksu.edu.sa (G.M.A.-S.)

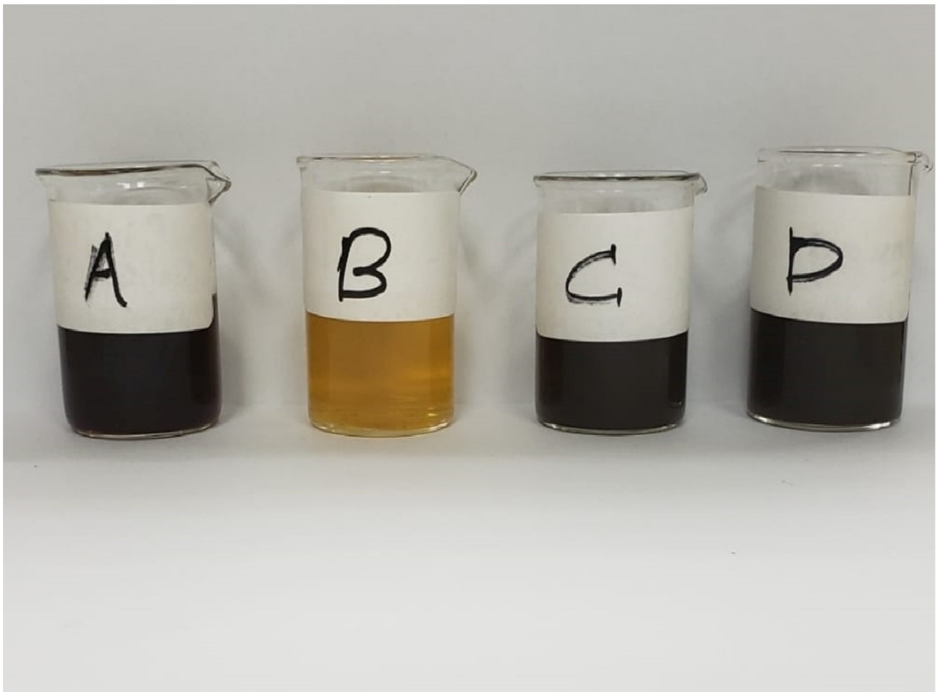

Figure. S1. *C. officinalis* stem bark methanol extract (**A**), iron oxide (**B**) and iron oxide nanoparticle solutions ((**C**) 50 mg extract + 0.1 M $FeCl_3$; (**D**) 100 mg extract + 0.1 M $FeCl_3$).

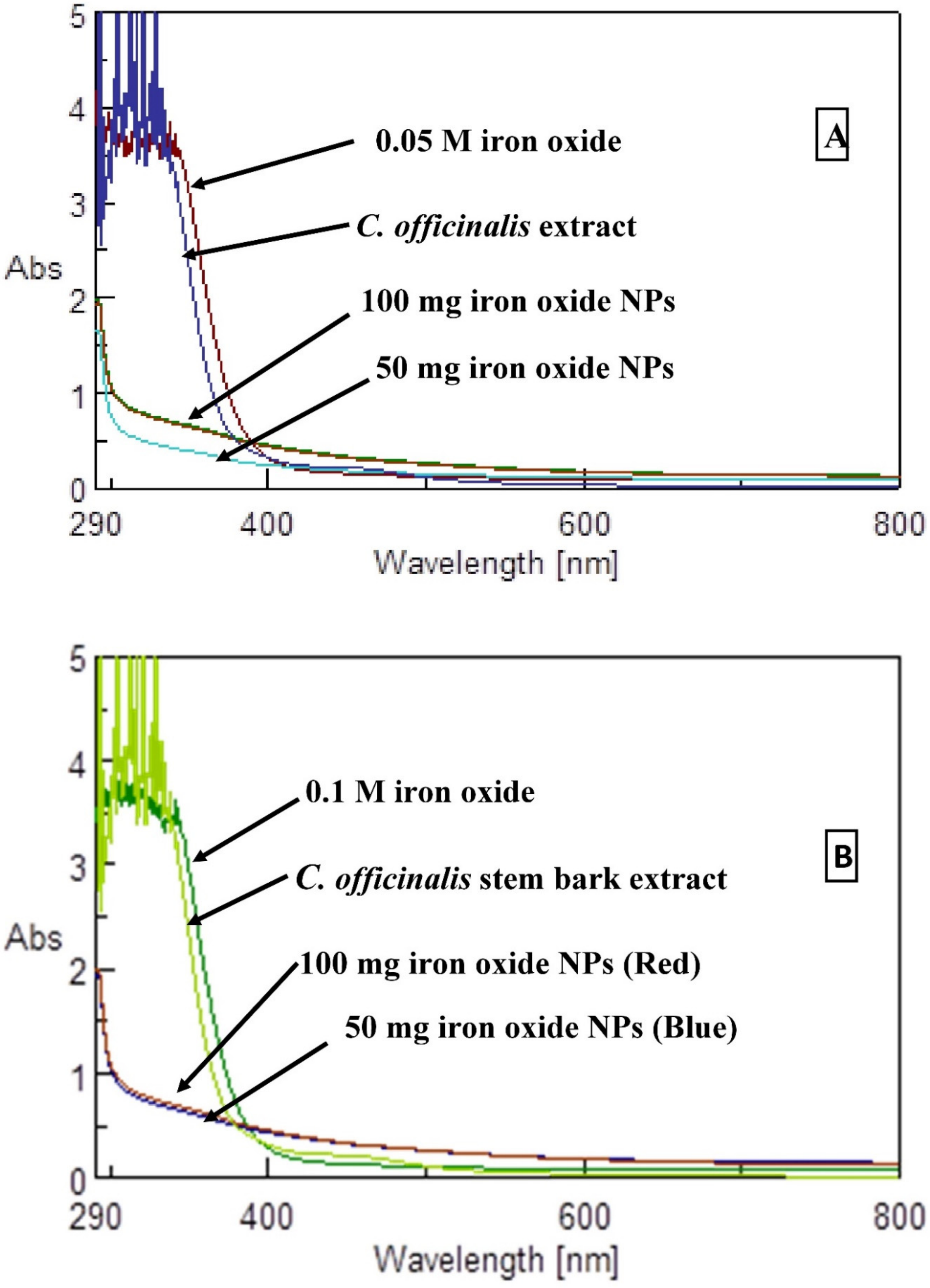

Figure S2. UV-vis spectra. Iron oxide NPs prepared by reacting 50 mg and 100 mg of *C. officinalis* stem bark methanol extract with 0.05 M (**A**) and 0.1 M (**B**) $FeCl_3$ solutions.

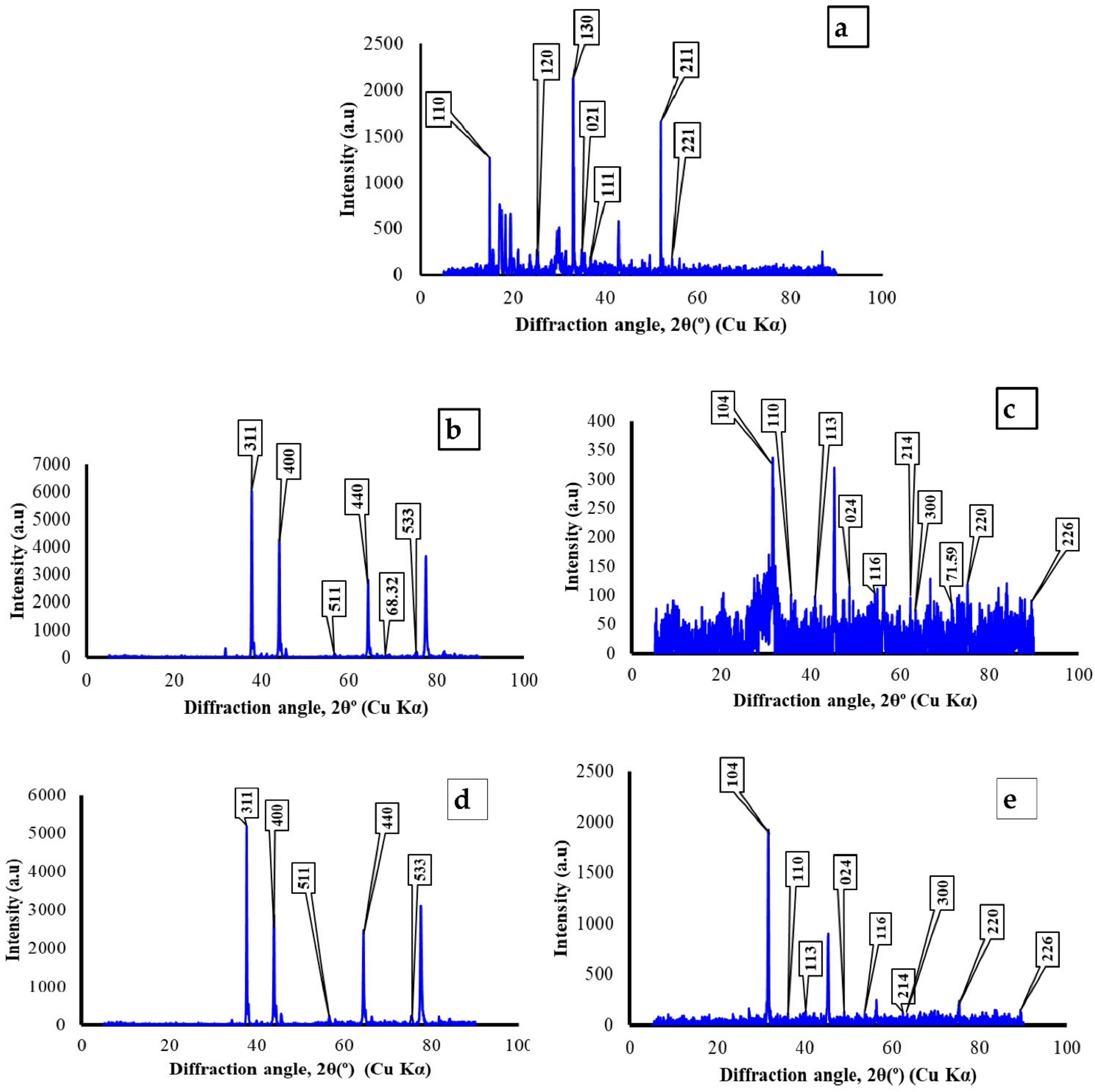

Figure S3. XRD spectra. Iron oxide (**a**). Iron oxide NPs prepared by reacting 0.05 M and 0.1 M $FeCl_3$ with 50 mg (**b**,**d**) and 100 mg (**c**,**e**) of *C. officinalis* stem bark methanol extract, respectively.

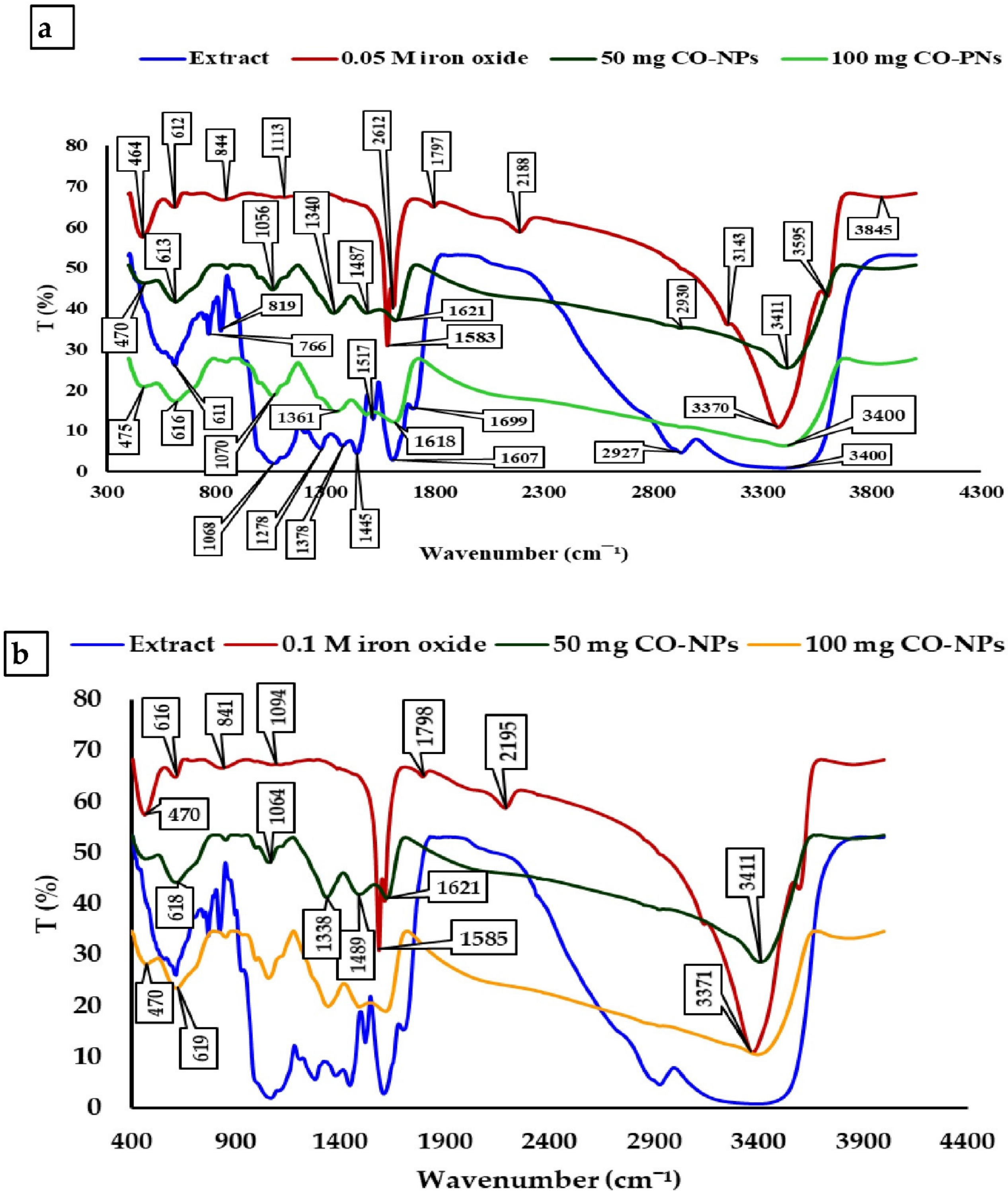

Figure S4. FT-IR spectra of *C. officinals* extract, iron oxide, and 50 mg and 100 mg NPs prepared using $FeCl_3$ at concentrations of 0.05 M (**a**) and 0.1 M (**b**).

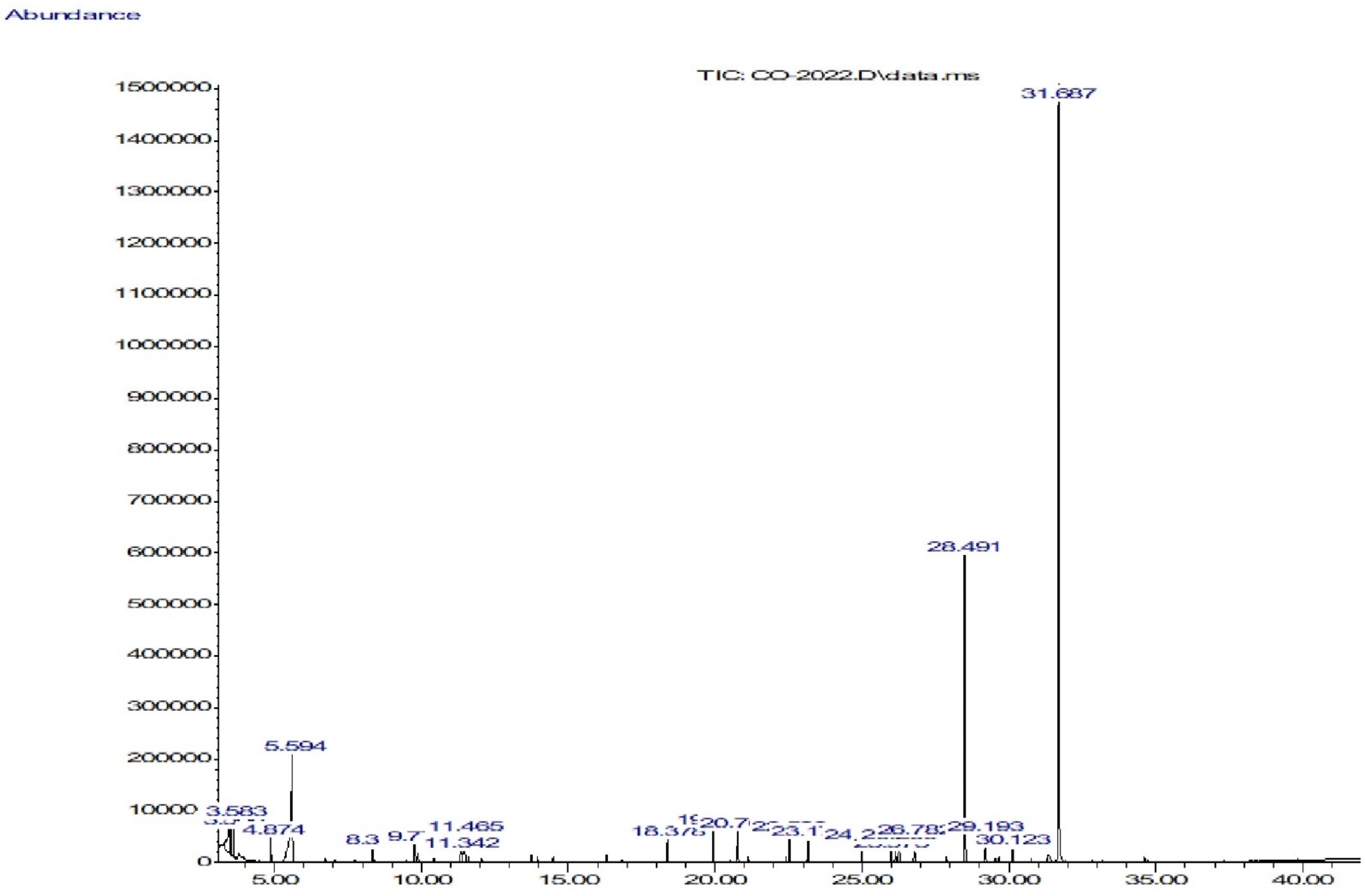

Figure S5. GC-MS chromatograms of the methanol extract components of *Cinchona officinals* stem bark.
